# Membrane proteome analysis identifies key components of sensing in *Phytophthora parasitica* zoospores

**DOI:** 10.1038/s41598-025-08701-z

**Published:** 2025-07-02

**Authors:** C. A. Lupatelli, A. Seassau, M. Magliano, M. L. Kuhn, A. Rey, M. Poët, L. Counillon, E. Evangelisti, P. Thomen, A. Attard, X. Noblin, E. Galiana

**Affiliations:** 1 Université Côte d’Azur, INRAE, CNRS, ISA, Sophia Antipolis, France; 2 Université Côte d’Azur, CNRS UMR 7010, Institut de Physique de Nice, Nice, France; 3 Université Côte d’Azur, CNRS, Laboratoire de Physio Médecine Moléculaire (LP2M), Nice, France

**Keywords:** Zoospores, Membrane, Proteome, Signal perception, Pathogens, Proteomics

## Abstract

*Phytophthora* plant pathogens rely on motile biflagellated zoospores to efficiently locate and colonise host tissues. While rhizospheric signals guiding zoospore movement toward roots are known, the protein composition of membranes mediating these responses remains unclear. Here, we used liquid chromatography with tandem mass spectrometry (LC–MS/MS) and proteomic data mining to analyse membrane fractions from the flagella and cell bodies of *Phytophthora parasitica* zoospores. Major classes of membrane proteins (receptors, transporters and enzymes) were identified and their subcellular distribution between flagella and cell bodies quantified. Immunolocalization revealed that while most membrane proteins are evenly distributed, a subset localizes to the flagella, suggestive of specialized roles in sensing and movement regulation, particularly for sterol recruitment and ion flux variations. These findings advance our understanding of protein-mediated dispersal and host targeting by zoospores and support the hypothesis that zoospores use polarized signal perception mechanisms for environmental sensing and movement.

## Introduction

Microbial species integrate a variety of environmental signals that shape their distribution and ultimately determine the microbiome composition within a given biotope. For pathogenic species, especially those in the oomycete class, responding to environmental factors is critical for exploring a microenvironment and establishing disease. Climatic forces, such as wind and water currents, are key to disseminating these pathogens, especially in soil and aquatic ecosystems^1^. Additionally, the chemical and physical properties of the biotope create conditions that can either facilitate or inhibit microbial exploration^2^. Within this complex environment, interactions between the microbiome and host plants play a crucial role, as host-emitted signals can stimulate chemotactic movement, trigger germination and sporulation, or initiate host penetration^2–4^.

In *Phytophthora* plant pathogens, the spread of disease in soil relies on motile zoospores that actively swim toward host roots^5,6^. Unicellular zoospores (6–10 µm diameter) propel themselves using two flagella (10–20 µm length) in a eukaryotic version of the 'run-and-tumble’ motion, where coordinated beats drive straight helicoidal movements, and directional shifts involve a brief posterior flagellum pause^7,8^. Morphologically, zoospores are polarized cells, with two distinct flagella: an anterior decorated with mastigonemes that enables directional thrust reversal, and a posterior with a whiplash structure^7,9,10^.

Zoospores employ multiple taxis mechanisms to detect and integrate environmental cues critical for host localization and infection. Positive rheotaxis and negative geotaxis enable *Phytophthora* species to swim against currents and remain near the soil surface^3^. Navigation is further guided by electrotaxis, chemotaxis, and ion currents generated by plant roots. For instance, *P. sojae* and *P. cinnamoni* zoospores are attracted to plant exudates^11,12^. Potassium ion gradients, generated by soil activities, also influence zoospore swimming and aggregation^13,14^. In addition, internal signalling from pioneer zoospores plays a role in aggregating *P. parasitica* zoospores^15^.

Several molecular mechanisms that enable zoospores to detect and adjust their movement in response to external signals have been previously identified^3^. Potential chemoreceptors include G-protein-coupled receptors (GPCRs). For example, *P. infestans* and *P. sojae* mutants that lack the Gα subunit exhibit disrupted swimming patterns and lose chemotaxis toward glutamic acid and daidzein, respectively^16,17^. Moreover, recent studies have clarified the roles of leucine-rich repeat receptor-like kinases (LRR-RLKs) in *P. sojae* isoflavone-mediated chemotaxis, identifying IRK1 as the receptor for genistein and IRK2 as a co-receptor that enhances ligand binding and regulates chemotaxis via phosphorylation of the G protein α subunit (PsGPA1)^18^.

The availability of *Phytophthora* genome sequences, coupled with advanced comparative omics approaches, raises the possibility for more systematic research into the perception and response of pathogen zoospores to signals in their environment.

In the present study, a proteomics approach was used to investigate the sensory repertoire of zoospores of *P. parasitica*, a polyphagous pathogen that infects a wide range of hosts^19^. We focused on three key questions: What is the plasma membrane (PM) protein repertoire that zoospores use to perceive and transduce external stimuli and navigate towards host plants? Do novel components within this repertoire reveal new insights into zoospore sensing? Are molecular sensors asymmetrically distributed between the flagella and the cell body, suggesting a specialization of roles?

To address these questions, we generated enriched membrane fractions from both the flagella and cell bodies of zoospores, to enable targeted analysis of these structures. Using liquid chromatography-tandem mass spectrometry (LC-MS/MS), the full zoospore membrane protein repertoire was qualitatively described and then the differences between the two fractions were quantified. Among the repertoire, we selected and characterized candidates such as enzymes, transporters, and receptors, investigated their subcellular localisation and used bioinformatics to investigate their putative roles in sensing and signal processing. Our findings shed light on the molecular basis of zoospore directed navigation and host signal detection.

## Materials and methods

### Isolation of flagella and cell bodies from zoospore suspensions

The mycelium of *P. parasitica* (=*P. nicotianae*^19^) was cultured in a V8 liquid medium (for 1 L: 800 mL distilled water, 200 mL V8 juice, 3 g CaCO_3_) and subsequently incubated on 2% water agar for 7 days to induce sporulation. Release of zoospores (10^6^ cells/mL) was achieved in water^13^. Flagella (F) were separated from cell bodies (CB) by vortexing zoospore suspensions for two minutes. The separation method was adapted from a previously described protocol^20^ with modifications to the number and speed of centrifugation steps. Bright-field microscopy was used to check the transition of swimming zoospores to non-motile cysts while fluorescence microscopy with Tubulin Tracker Green staining was used to visualize flagella and cell bodies in suspension. The CB fractions were pelleted by centrifuging the deflagellated-cell suspension (3000*g*, 5 min, RT). The supernatant was subjected to a second centrifugation (3000*g*) to remove any CB contamination. The F fractions were pelleted at 31,000*g* (30 min, 4 °C).

### Preparation of membrane-enriched fractions

We enriched four replicates of CB and F fractions in membrane (M) proteins using the MemPER Kit (ThermoFisher, Waltham, MA USA) and adapting the protocol for Suspension of Mammalian Cells. The CB and F pellet fractions were resuspended in 1 mL of the Cell Wash Solution buffer. After centrifugation at 1000*g* (3 min, RT) for CB fractions and 17,000*g* (15 min, 4 °C) for F fractions, the pellets were resuspended in the Permeabilization Buffer then the Solubilization Buffer. At each step F and CB pellets were resuspended in 200 µL and 500 µL of solution, respectively, incubated and gently mixed for 15 min at 4 °C, and centrifuged at 17000*g* (15 min, 4 °C). Supernatants, enriched in membrane fractions, were recovered in the Solubilization Buffer, and stored at − 80 °C. The protein concentration of the membrane-enriched fractions (M-F and M-CB) was determined by spectrophotometry (660 nm) using the Pierce™ BCA Protein Assay Kit with an ionic detergent compatibility reagent (ThermoFisher). The quality of fraction separation and membrane protein enrichment was confirmed by immunoblotting for the mastigoneme protein PPTG_02441, localized on the membrane of the anterior flagella.

### Liquid chromatography and mass spectrometry analysis

The method is detailed in Supplementary Data 1. Briefly, four biological M-F and M-CB replicates were fractionated by electrophoresis through SDS-polyacrylamide (10%) gels, stained with Coomassie blue and sectioned into several bands (1 mm^3^) (Supplementary Data 1). Each band was subjected to an acetonitrile washing, reduced with 10 mM DTT in 50 mM N_4_HCO_3_ for 30 min at 56 °C, and alkylated with 55 mM iodoacetamide in 50 mM NH_4_HCO_3_ for 20 min at 25 °C in the dark. The proteinaceous content of bands was digested overnight at 37 °C in a solution containing 25 mM NH_4_HCO_3_, 5 mM CaCl_2_, and 12.5 ng/µL sequencing-grade modified trypsin. The resulting peptides were extracted with acetonitrile. The samples were dried and desalted using OMIX C18 pipette tips (100 µL, A57003100,Agilent, Santa Clara, CA, USA).

Samples were analysed using a nanoUHPLC system (nanoElute) coupled to a TimsTOFpro mass spectrometer (Bruker Daltonics, Germany). Each sample (10 µL) was separated on a reverse-phase C18 column with an integrated CaptiveSpray Emitter (75 µm ID × 250 mm, 1.6 µm, Aurora Series with CSI, ionOpticks, Australia). The TimsTOFpro mass spectrometer was operated with the CaptiveSpray nano-electrospray ion source. MS and MS/MS data were acquired in positive polarity using the Parallel Accumulation-Serial Fragmentation (PASEF) Data Dependent Acquisition (DDA) mode. Peptides were detected over a mass range of 100 to 1700 m/z, with a target intensity of 20,000 and an intensity threshold of 2500. The acquired DDA spectra were initially examined using Data Analysis software (version 5.3, Bruker Daltonics, Germany). Subsequently, the data were analysed against the proteome predicted from the *P. parasitica* 310 genome with PEAKS Studio (version Xpro, Bioinformatics Solutions)^21^. Only proteins identified with an FDR of 1%, with at least one unique peptide, were selected.

The relative abundances of the proteins in the M-F and M-CB replicates were measured using the PEAKS Q label-free quantification method. Only proteins with an FDR of less than 1% and a fold change greater than 2 were selected. To be considered for quantification, a minimum of one peptide was required, and peptides had to be identified in both groups and detected in at least two samples per group.

### Data analysis: identification and quantification

The initial protein identification list generated with PEAKS (6516 proteins) was refined. Proteins were retained if they were identified by at least two MS/MS spectra across all four biological replicates, whether from the cell body or flagella fractions. The reduced protein list was further refined using multiple prediction tools to identify membrane-associated and secreted proteins: TMHMM for transmembrane domains^22^, the NetGPI GPI for GPI-anchored proteins^23^ and the SignalP 5.0 for signal peptides^24^. The final list of 1069 proteins included those predicted to contain transmembrane domains, GPI anchors, and/or signal peptides. This set therefore comprised integral membrane proteins as well as peripheral or secreted proteins that may associate with membranes via lipid modifications or protein-protein interactions. The protein families, names and Gene Ontology (GO) annotations were obtained from the UniProt database^25^. Classification of membrane proteins into three main categories (receptors, transporters and enzymes) was performed as described by Almén et al.^26^ and by finding functional annotations in the Transporter Classification DataBase (TCDB)^27^, the Enzyme classification (EC) system and other references in the literature^28^.

### *In silico* functional and structural annotation

Proteins of interest were functionally annotated with UniProt and SMART^29^ to identify the protein family and domain architecture. When available, predicted models of 3D structures were retrieved from AlphaFoldD or generated through AlphaFoldD2^30^. Phyre2 was used to identify and retrieve the best-aligning crystal structures of the proteins of interest^31^. At the same time, Chimera was used for structural alignment and calculation of the root-mean-square deviation (RMSD)^32^. To cluster members of a specific protein family, sequences were retrieved from GenBank using a three-step process: (i) initial homology searches, against the predicted proteome of *Phytophthora parasitica*, were conducted using BLASTP (threshold 1e-06) followed by (ii) searches against other public predicted proteomes from the REFSEQ_protein databank. (iii) Sequences were analysed with the CDvist tool^33^ to search for protein multidomains, selecting sequences that displayed consistent patterns and orders of domain organization and exhibited at least 25% similarity to each other. Phylogenetic analysis was conducted using the Phylogeny.fr platform (https://www.phylogeny.fr/phylogeny.cgi) to assess evolutionary relationships and support homology between *P. parasitica* tyrosine kinases and their counterparts in related species. Protein sequences from *P. parasitica, P. sojae* and *P.infestans* were aligned using MUSCLE, followed by tree construction with PhyML, incorporating the aLRT statistical test for branch support^34,35^. The resulting tree was visualized using TreeDyn^34^.

### Immunolocalization experiments

Immunohistochemistry was performed with commercially produced rabbit polyclonal antibodies targeting peptides for PPTG_02441 (CGNPGRLRTPEIAS), PPTG_09633 (CQDEYNDTIPTGGD), PPTG_07417 (CTGYGRNPTSWSLNDEN) and PPTG_13136 (CDLDNANTDGPLTEYLTKDA). Zoospores (concentration 5 × 10^5^ cells/mL) were first fixed using 1% (V/V) glutaraldehyde for 2 h at 4 °C, washed in phosphate-buffered saline (PBS) and treated with a blocking solution containing 3% bovine serum albumin in PBS, pH 7.2, for 30 min. They were then incubated for 3 h at 4 °C with each primary antibody, diluted 1:100, washed three times with PBS and incubated for 1h at RT with a FluoProbes 547H Donkey Anti-Rabbit IgG diluted 1:200 (Interbiotech – BioScience Innovations). Samples were rinsed three times and mounted in Fluoroshield (Sigma, St. Louis, MA USA). Image acquisition was performed using a Zeiss LSM 880  confocal laser scanning microscope (Carl Zeiss Microscopy, Germany).

For immunoblotting, samples (2 µg) were separated in 4–20% precast polyacrylamide gels (Bio-Rad), transferred onto a nitrocellulose membrane and stained with Ponceau red. The membranes were incubated and washed sequentially in PBS in the presence of 5% (w/v) nonfat dry milk, a primary rabbit polyclonal antibody (1:2000), a goat peroxidase-conjugated IgG directed against rabbit immunoglobulins (1:5000), and a chemiluminescent substrate for peroxidase. The bound antibodies were detected using the FusionX apparatus (Vilber, Marnela-Vallée, France) and the ECL Western Blotting Substrate protein labelling kit (Promega).

### Microscopy

Confocal laser scanning microscopy was performed on a Zeiss LSM 880 system equipped with a photomultiplier tube and four lasers: diode laser (405 nm, 30 mW), argon laser (458, 488, 514 nm, 25 mW), DPSS laser (561 nm, 20 mW), and He–Ne laser (633 nm, 5 mW). Imaging was conducted using a C-Apochromat 63×/1.2 W Corr objective and controlled via the ZEN 2 software suite. All imaging was conducted at the Microscopy Platform of the Sophia Agrobiotech Institute (INRAE 1355, UNS, CNRS 7254, INRAE PACA, Sophia Antipolis, France). Samples were mounted on 10-well specialty slides (Epredia, Thermo Fisher Scientific, New Hampshire, USA). Images from immunolocalization experiments were acquired using a 561 nm laser, with excitation at 561 nm and emission at 629 nm. Tubulin Tracker Green labelling was visualized using a 488 nm laser, with excitation at 488 nm and emission at 522 nm.

## Results and discussion

### Production of cell body and flagella membrane protein-enriched fractions

**Fig. 1:**
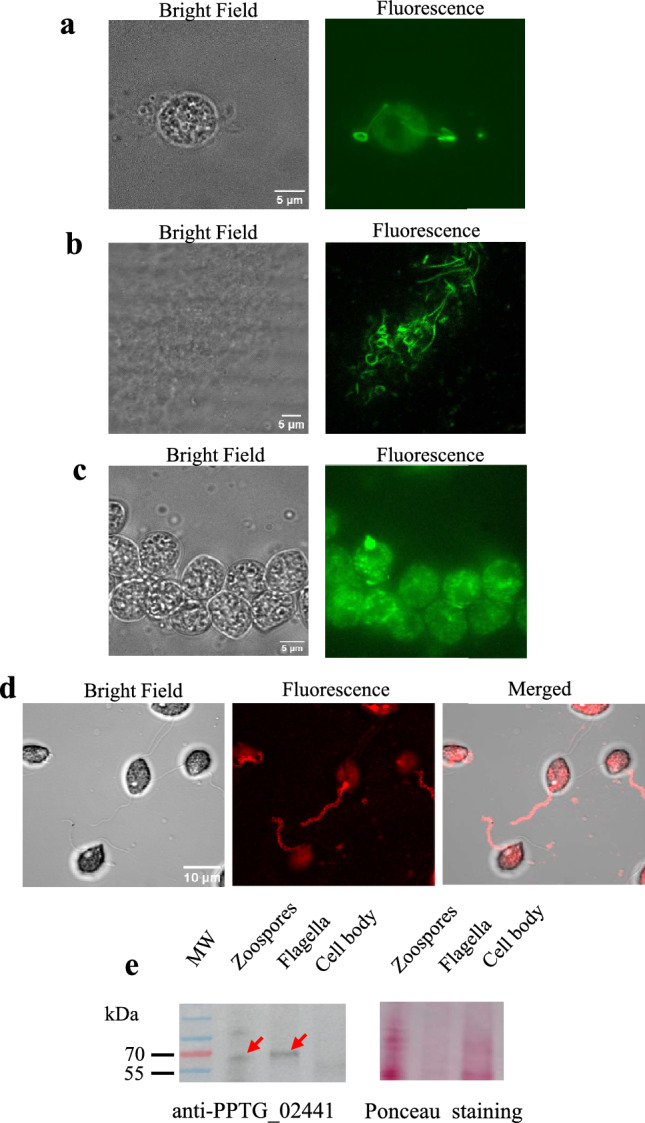
Cell body and flagella membrane-enriched fractions. (**a**–**c**) Bright field (left) and Fluorescence (right) views of Tubulin Tracker Green labelling of an entire zoospore, separated flagella and cell body fractions. Images acquired with a Zeiss LSM 880 confocal microscope. (**d**) Bright field (left), Fluorescence (center) and Merged (right) views of zoospores labelled with antibodies against PPTG_02441. Images acquired with a Zeiss LSM 880 confocal microscope. (**e**) Immunoblot analysis using the antibody against the mastigoneme protein, PPTG_02441, which is detected at the expected calculated molecular weight (64 kDa) (left). Red arrows indicate the corresponding bands. Ponceau staining (right) indicates protein loading. Original immunoblot and red Ponceau staining are presented in Supplementary Fig. 2.

To investigate the overall composition and distribution of zoospore membrane proteins, we purified cell body (CB) and flagellar (F) fractions from swimming *P. parasitica* zoospores (Fig. 1). The homogeneity of the purified fractions was assessed using light microscopy and fluoresecent tubulin staining (Fig. 1a–c). The F fraction comprised elongated hair-like and curved structures observed in detached flagella (Fig. 1b, Supplementary Fig. 1a), while the CB fraction comprised multiple round cells typical of encysted zoospores (Fig. 1c, Supplementary Fig. 1b). We further processed the F and CB fractions to obtain the membrane protein-enriched fractions M-F and M-CB. As a quality control, we analysed M-F and M-CB by immunodetection using a polyclonal antibody raised against the mastigoneme protein PnMas1 (PPTG_02441), which localizes to the anterior flagellum of zoospores. The antibody labelled the anterior flagellum and partially the cell body at the flagellum base (Fig. 1d), likely corresponding to the diffusion barrier regulating protein migration toward the flagellar structure^36,37^. Western Blotting revealed a single band at 64 kDa corresponding to the predicted molecular weight of PPTG_02441, in samples corresponding to intact zoospores (used as a positive control) and the M-F fraction, but not in the M-CB fraction (Fig. 1e). Together, these results confirm the efficiency of our extraction method and the purity of the M-F and M-CB fractions.

### Membrane-associated proteins related to zoospore biology

**Fig. 2:**
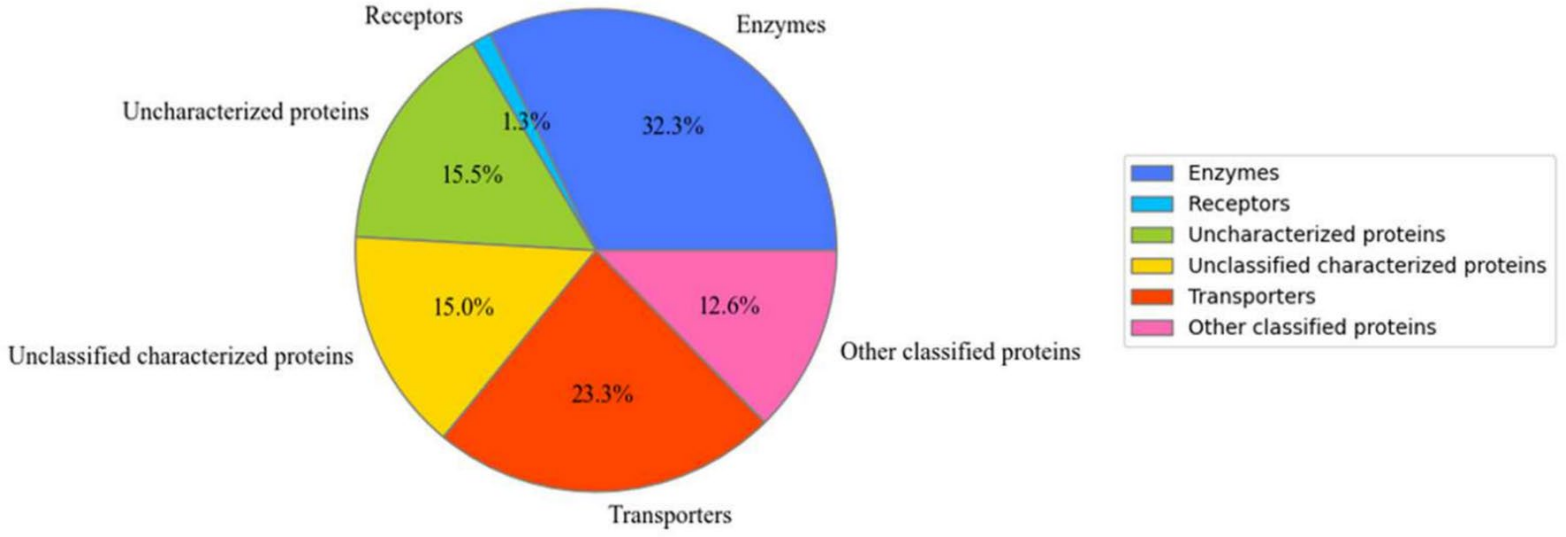
Categories of membrane proteins identified in zoospores. Membrane proteins were classified into three major functional categories: transporters, enzymes, and receptors. Proteins involved in sensing or relevant to *Phytophthora* biology but not fitting these primary categories were designated as ‘Other classified proteins’. Proteins with unrelated functions were excluded from further analysis. Notably, 15% of the identified membrane proteins lacked any functional annotation .

LC-MS/MS analysis of the M-F and M-CB fractions identified 1069 membrane-associated proteins (Fig.2) (Supplementary Dataset File 1). Of these, 608 proteins (~60%) were confidently annotated as transporters, receptors, or enzymes—core functional classes typically involved in membrane dynamics^26^. The remaining proteins were grouped into three categories based on their annotation status: (i) Uncharacterized proteins, which lacked any functional annotation; (ii) Other classified proteins, which were annotated and assigned to defined functional categories outside of transporters, enzymes, or receptors, and were considered relevant to *Phytophthora* biology or potential sensing functions; and (iii) Unclassified characterized proteins, which had some annotation but could not be confidently assigned to any main membrane protein class (transporter, enzyme, or receptor), nor to the other classified group, due to lack of relevance to sensing or *Phytophthora*-specific processes. For downstream analyses, we focused on transporters, receptors, enzymes, and other classified proteins, as these groups were more likely to include candidates involved in zoospore sensing, signal transduction, and motility regulation. Proteins in the uncharacterized and unclassified characterized groups were excluded due to insufficient or irrelevant functional information.

#### Enzymes

**Fig. 3:**
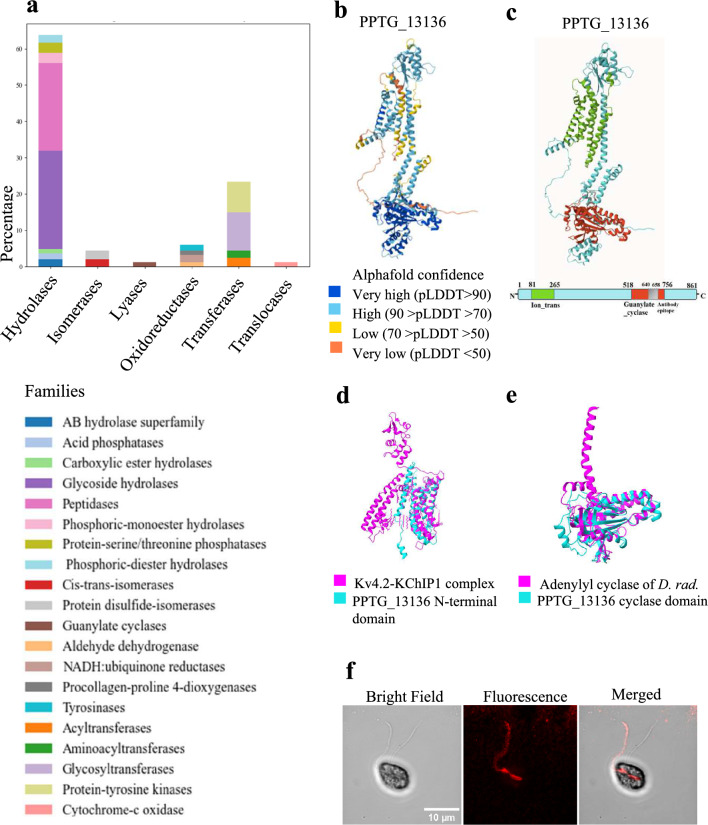
Zoospore membrane enzymes. (**a**) Distribution of enzyme families identified through membrane proteomic analysis of zoospores, presented as percentages. (**b**) AlphaFold model of PPTG_13136 with the pLDDT score indicating the accuracy of the prediction for each residue. (**c**) Domain architecture of PPTG_13136 indicating the position of the Ion_trans PFAM (green) and Guanylate cyclase domains (red). The same architecture was established for PPTG_13761. (**d**) Local alignment using ChimeraX of the 3D-structure prediction of the PPTG_13136 (cyan) N-terminal domain with a segment of the CryoEM structure of the human voltage-gated potassium channel, Kv4.2-KChIP1 complex (magenta, PDB: 2R9R). RMSD 12. (**e**) Superposition of the PPTG_13136 nucleotide cyclase domain (cyan) and Chain B of photoactivated adenylyl cyclase of *Deinococcus radiodurans* (magenta, PDB: 6FHT). RMSD 4. (**f**) Bright-field (left), Fluorescence (center) and Merged (right) confocal laser microscopy images of zoospores stained for PPTG_13136.

Enzymes constituted the largest category, accounting for 32% of the classified proteins (Fig. 3a). Hydrolases made up more than 50% of identified enzymes, including 67 glycoside hydrolases (GH), with 9 cellulases and 60 peptidases. Classification of carbohydrate-active enzymes^38^ and *in silico* analysis of genes encoding cell wall-degrading enzymes in the *P. parasitica* genome^39^ revealed that the zoospore GH repertoire spans eleven hydrolase families (GH1, GH5, GH17, GH30, GH31, GH35, GH38, GH47, GH63, GH72, GH81), highlighting its capability to degrade various carbohydrates. Members of the GH1 family are thought to modify the *P. parasitica* cell wall rather than that of the host^39^. Members of the GH30 family (PPTG_08507, PPTG_08509) were annotated as glucosylceramidases and may play specialized roles in cellular recognition processes by modulating variations in the glucosylceramide head groups of their substrates, similar to functions observed in other eukaryotes^40^. Among the 60 peptidases identified, significant functional diversity was observed. Some were classified as metallopeptidases belonging to the thimet oligopeptidase family^41^ (e.g., PPTG_03858), which are known in *Trypanosoma brucei* for their role in the generation of quorum sensing signals^42^ and so are potentially involved in sensing.

Alongside hydrolases, 25% of the enzymes were identified as transferases, including 21 tyrosine kinases and 26 glycosyltransferases. The glycosyltransferase group, including β-1,3D-glucan- (PPTG_08579, PPTG_13258, PPTG_13182) and cellulose- (PPTG_17902) synthases, may constitute preassembled enzymatic machinery that rapidly constructs the primary cyst wall post-deflagellation^43^. The 21 proteins annotated according to UniProt as tyrosine kinases exhibited characteristic sensing properties, with a typical organization reminiscent of the family of receptor-like kinases (RLKs): an N-terminal extracellular domain, a transmembrane domain, and a C-terminal kinase domain (KD)^44^. Notably, six of these (PPTG_00076, PPTG_01724, PPTG_01987, PPTG_09207, PPTG_14643, PPTG_16352) are distinguished by one to three Leucine Rich Repeat (LRR) domains in their extracellular region, which are crucial for protein binding and upstream signalling pathway activation^45^. Although our annotation pipeline classifies all six proteins as tyrosine kinases with predicted enzymatic activity, mounting evidence—particularly from *Phytophthora sojae*—indicates that LRR-RLKs often function as extracellular sensors mediating environmental and host-derived signal perception^18,46^. To investigate potential functional parallels, we performed BLAST analyses, revealing strong sequence homology between these *P. parasitica* RLKs and characterized *P. sojae* counterparts—namely PsRLK23 (UWU45076.1), PsRLK13 (UWU45070.1), PsRLK18 (UWU45072.1), PsRLK12 (UWU45060.1), PsRLK6 (UWU45069.1), and PsRLK10 (UWU45058.1)—all implicated in environmental sensing^47^. Phylogenetic analysis (Supplementary Fig. 3) provides additional support for the observed homology. These findings suggest that *P. parasitica* RLKs may similarly mediate extracellular signal detection. Nonetheless, functional assays in *P. parasitica* are essential to confirm these roles and clarify their contribution to signal transduction pathways.

Finally, a small subset of lyases (2% of the enzymes) were identified as guanylate/adenylate cyclases (GCs/ACs), including PPTG_13136, PPTG_13761, and PPTG_08439, which are potentially crucial for zoospore sensing and signalling by catalysing cyclic GMP (cGMP) or cyclic AMP (cAMP) synthesis^48^. GCs and ACs are classified into soluble and membrane-bound types, with the former involved in signalling pathway functions like protein kinase activation and the latter categorized based on ligand specificity^49^. The proteins PPTG_13136 and PPTG_13761 annotated in the *P. parasitica* genome as voltage-gated ion channels, were found to be unique members of a bimodal membrane-bound protein family, presenting similarities with no other protein of *P. parasitica*. They share 38.75% sequence identity and each has two structurally independent domains. The N-terminal domain (PPTG_13136: residues 81-265; PPTG_13761: residues 81-226) includes an Ion_trans PFAM domain, characteristic of sodium, potassium, and calcium ion channels (Fig. 3b,c). These domains feature 5 and 3 transmembrane helices respectively, with segments containing typical motifs of the voltage sensor S4 segment (VSD) in voltage-dependent gated channels (Supplementary Fig. 4a)^50^. Homology searches revealed partial similarity to human voltage-gated potassium channel subunits, and 3D structural predictions aligned these segments with the shaker family voltage-dependent K⁺ channels and the Kv4.2-KChIP1 complex (Fig. 3d). However, both proteins lack homology in the pore helix region essential for K⁺ channel gating. The second domain, encompassing residues 518–756 of PPTG_13136 and 533–774 of PPTG_13761, was annotated with a Guanylate_cyc PFAM domain (Fig. 3c and Supplementary Fig. 4b). In addition, Phyre2 modelling predicted these regions to adopt 3D structures resembling guanylate and adenylate cyclases (Fig. 3e), suggesting a potential signalling function. Given the absence of close structural homologs with experimentally resolved structures to date, these models should be viewed as exploratory frameworks for a novel, bimodal protein family, seemingly restricted to Stramenopiles and Alveolates (Supplementary Dataset File 2; Supplementary Fig. 4c,d). Taken together, the organization into two domains comprising a voltage sensor S4 segment (VSD) and a guanylyl cyclase (GC)/adenylyl cyclase (AC) suggests two potential functions for PPTG_13136 and PPTG_13761. They may act as ion channels with an enzymatic domain that plays a regulatory role, similar to certain plant cyclic nucleotide-binding domain (CNBD) superfamily ion channels, also found in ciliates and green algae^51^. Alternatively, they might act as ion current-regulated enzymes. This is akin to a novel adenylyl cyclase identified in *Paramecium* species, which features a VSD and a K^+^ channel pore localized at the ciliary level^52^, or the voltage-sensing phosphatases (VSPs) in choanoflagellates, where a VSD triggers phosphatase activity^53^. As mentioned above, we found similar bimodal proteins to be exclusively present in at least 90 other flagellated or ciliated organisms (Supplementary Dataset File 2), suggesting that PPTG_13136 and PPTG_13761 might have a role also in motility-related processes. Supporting this hypothesis, immunolocalization revealed PPTG_13136 on *P. parasitica* zoospores, predominantly on a single flagellum, and possibly at its base (Fig. 3f). Further functional characterisation of these proteins is essential to elucidate their roles and regulatory mechanisms. Their conservation across several *Phytophthora* species (Supplementary Dataset File 2) could enable targeted manipulation using CRISPR-Cas9 in genetically tractable models such as *P. sojae*^54^**.** This includes gene knockouts, mutant phenotyping, and expression profiling to investigate their roles in sensing-related processes. Complementary approaches, such as heterologous expression, could further support functional analysis.

#### Transporters

**Fig. 4:**
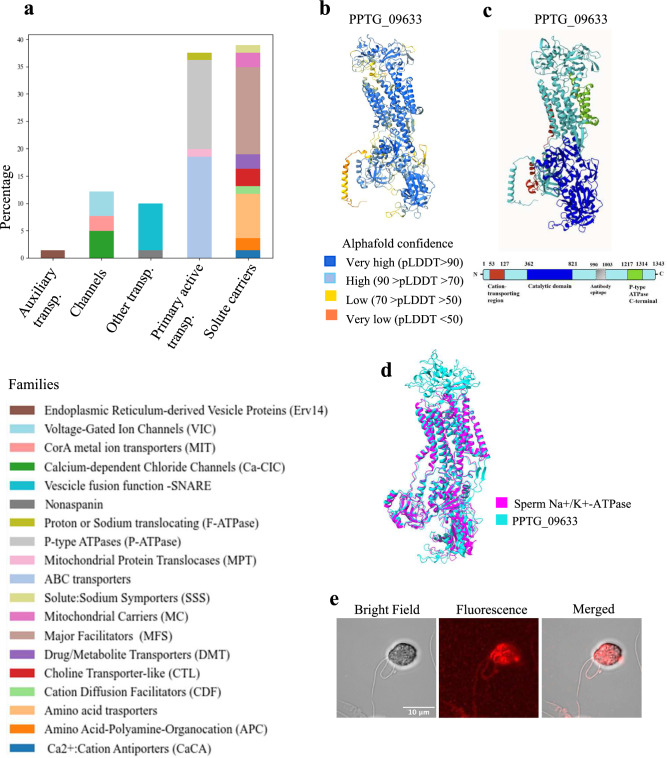
Zoospore membrane transporters. (**a**) Distribution of transporter families identified through membrane proteomic analysis of zoospores, presented as percentages. (**b**) AlphaFold model of PPTG_09633 with the pLDDT score indicating the accuracy of the prediction for each residue. (**c**) Schematic representation of PPTG_09633 domain architecture. The protein includes a cation-transporting P-type ATPase Nterminal region (53–127 aa) coloured in red, a catalytic domain (362–821 aa) in blue, and a P-type ATPase C-terminal region (1217–1314 aa) in green. (**d**) Tight structural alignment between the predicted 3D structure of PPTG_09633 (cyan) and the crystal structure of human sperm Na^+^/K^+^-ATPase (magenta, PDB: 8ZYJ). RMSD 5. (**e**) Bright-field (left), Fluorescence (center) and Merged (right) confocal laser microscopy images of zoospores stained for PPTG_09633.

Transporters were the second-largest heterogeneous category in the zoospore membrane proteome (23% of the total, n = 249; Fig. 4a). Similar patterns have been previously observed in other flagellate species such as *Leishmania major*, *Trypanosoma brucei* and *Trichomonas vaginalis*, which have 247, 299 and 408 transporters, respectively^55^. This underscores the pivotal role of transporters in mediating the selective passage of molecules across the membrane of flagellated cells, thereby maintaining the electrochemical gradient essential for energy production and metabolic exchange, in particular in all aspects of the motility process, including environmental sensing, force generation and signalling^56^.

Within this category, solute carriers comprised 38% of transporters, mainly from the major facilitator superfamily (n = 35). Primary active transporters were similarly prevalent, representing 33% of the transporters, including P-type ATPases (n = 36) and ABC transporters (n = 41), along with a smaller subset of F-type ATPases (n = 3). Among these, two subunits of the V-type proton ATPase were identified: the integral membrane alfa subunit (PPTG_05924) and the proteolipid subunit (PPTG_11905), which in zoospores localize to the spongiome membranes of the water expulsion vacuole (WEV), facilitating osmoregulation by powering water expulsion^57^.

Among the transporters, our analysis focused on the sodium-potassium ATPase, PPTG_09633, as it was the most abundant protein among all the 1069 identified membrane proteins (Fig. 4b). Eukaryotic Na^+^/K^+^-ATPases operate in the PM to maintain cellular homeostasis by transporting three Na^+^ ions out and two K^+^ ions into the cell, against their concentration gradients. These enzymes consist of an α-subunit with 10 transmembrane regions, which houses the catalytic and ion-binding sites, and a smaller β-subunit, which aids in membrane targeting and K^+^ ion access^58^. PPTG_09633 exhibited α-subunit characteristics (Fig. 4c) and *in silico* annotation associated it with K^+^ import and Na^+^ export functions across the PM. By screening the *P. parasitica* genome, two additional Na^+^/K^+^-ATPase α-subunits were identified, both of which were included in the list of membrane proteins. This finding aligns with estimates for *P. infestans*, which also presents two predicted Na^+^/K^+^-ATPase α-subunits^59^. No β-subunits were found, consistent with previous studies indicating their absence in fungi and most Protista^60^. The function of Na^+^/K^+^-ATPases in *Phytophthora* remains to be elucidated. Previous studies on *Pythium* species indicate how a putative P-type ATPase may pump K^+^ into the cell in low K^+^ environments^59^. In higher eukaryotic flagellate species, Na^+^/K^+^-ATPase functions have been widely characterized and are also associated with regulation of sperm flagellar beating^61–63^. Comparative alignment revealed high sequence conservation between PPTG_09633 and the mammalian sperm Na^+^/K^+^-ATPase isoform α4, with similar 3D-structural alignment, suggesting functional similarities (Fig. 4d). Moreover, PPTG_09633 was immunolocalized on both flagella and cell bodies (Fig.4e), showing that the Na^+^/K^+^-ATPase is distributed throughout the zoospore, contributing both to overall cell homeostasis and, on the flagella, potentially playing a role in motion regulation similar to the sperm Na^+^/K^+^-ATPase.

Finally, the results highlighted the critical function of pore-mediated membrane transport, demonstrated by the presence of diverse voltage-gated ion channels (n = 10), comprising 14% of transporters and including a putative potassium channel (PPTG_12381). Voltage-gated channels are indispensable in flagellate behaviour, as demonstrated in *Chlamydomonas*, where specific voltage-gated calcium channels regulate photobehavioural responses^64^, and in human spermatozoa, where the CatSper2 voltage-gated calcium channel drives hyperactivation and vigorous flagellar movements, enabling sperm to penetrate the egg cell membrane during fertilization^65^. However, none of the *P. parasitica* proteins annotated as calcium channels (PPTG_16517, PPTG_13534, PPTG_11048) showed similarities to CatSper2.

#### Receptors

**Fig. 5:**
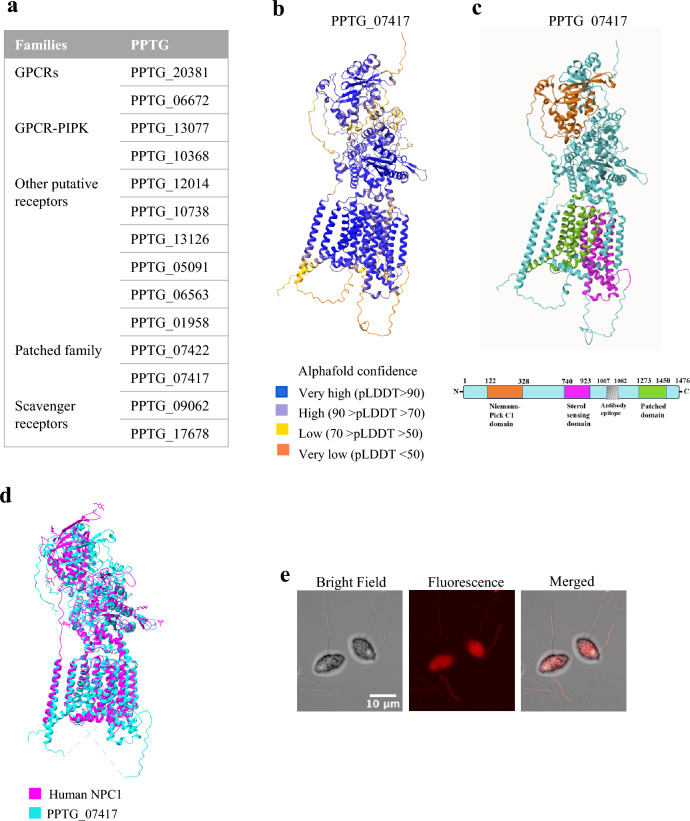
Zoospore membrane receptors. (**a**) Receptors identified through membrane proteomic analysis of zoospores. (**b**) AlphaFold model of PPTG_07417 with pLDDT score indicating the accuracy of prediction for each residue. (**c**) Domain structure of PPTG_07417, positioning the Niemann-Pick C1 domain (122–328 aa) coloured in orange, Sterol Sensing domain (740–923 aa) in magenta, and Patched domain (1273–1450 aa) in green. (**d**) Structural alignment of PPTG_07417 structure (cyan) with the crystal structure of fulllength human NPC1 (magenta, PDB: 3JD8). RMSD = 14. (**e**) Bright-field (left), Fluorescence (center) and Merged (right) confocal laser microscopy images of zoospores stained for PPTG_07417.

Receptors represent only 1.3 % (n = 14) of the identified membrane proteins (Fig. 5a).

Interestingly, we found only a few of the well-known components of major signalling pathways of microbial eukaryotic cells, such as G-protein-coupled receptors (GPCRs) and Patched family receptors from the Hedgehog (Hh) pathway^66^. While the human genome is estimated to encode approximately 950 GPCRs, of which 500 are odorant or taste receptors^67^, the genome of microorganisms such as *Dictyostelium* only encodes 55 GPCRs. Similarly, *Phytophthora* species (*P. infestans, P. sojae, P. ramorum*) harbour > 50 GPCRs, 12 of which are fused to a phosphatidylinositol phosphate kinase (PIPK) domain^68^. One GPCR-PIPK proved to be involved in spore germination, hyphal elongation, sporangia cleavage and infection^69^. In our results, only two zoospore membrane proteins were identified as GPCRs (PPTG_20381 and PPTG_06672) and two as specific GPCR-PIPKs (PPTG_13077, PPTG_10638). This suggests that GPCRs may be less prevalent in the zoospore membrane so other sensory proteins likely contribute to the overall sensing capabilities.

Next, we identified two proteins, PPTG_07422 and PPTG_07417, that contain a sterol-sensing domain (SSD) and are involved in sterol signalling in *Phytophthora*^70^. SSD domains, approximately 180 amino acids in length, play a crucial role in the sensing of sterol substrates. These domains are found in proteins involved in sterol signal transduction, including sterol carrier proteins (SCP) like Niemann-Pick disease C1 (NPC1), essential for intestinal cholesterol absorption, and Patched (PTC) proteins^70,71^. *Phytophthora* species are unable to synthesize sterols and may use these receptors to get sterols from their environment and support their growth^71^. Four *P. capsici* genes, including homologs of PPTG_07422 and PPTG_07417, which encode sterol-sensing domain (SSD)-containing proteins, have been shown to play important roles in asexual reproduction and pathogenicity^70^. Similarly, in *P. cinnamomi*, four PcSCP genes—homologous to the proteins characterized in our study—resulted crucial for sterol recognition and acquisition, promoting both mycelial growth and sporangial formation under exogenous sterol conditions^72^. The identification of two proteins with SSDs on zoospore membranes raises the question of how zoospores contribute to *Phytophthora* detection and uptake of sterols, mechanisms that have only recently been explored. Remarkably, PPTG_07417, the protein with a higher number of unique identified peptides, showed an N-terminal NPC1-like domain, an SSD and a patched receptor domain (Fig. 5b,c). BLAST and 3D structural analyses revealed highly significant alignment between PPTG_07417 and NPC1 throughout their entire structures, especially within the SSD (Fig. 5d). These findings corroborate the study by Wang* et al*.^70^ suggesting a notable degree of evolutionary conservation of the NPC1 family. While SSD proteins in *P. cinnamomi* are involved in sterol acquisition during later stages^72^, the localization of PPTG_07417 to the flagella and cell body (Fig. 5e), suggests that its functional role may not be active during early zoospore stages but rather becomes critical as the organism progresses into sterol-dependent phases of development.

#### Other classified proteins

**Fig. 6:**
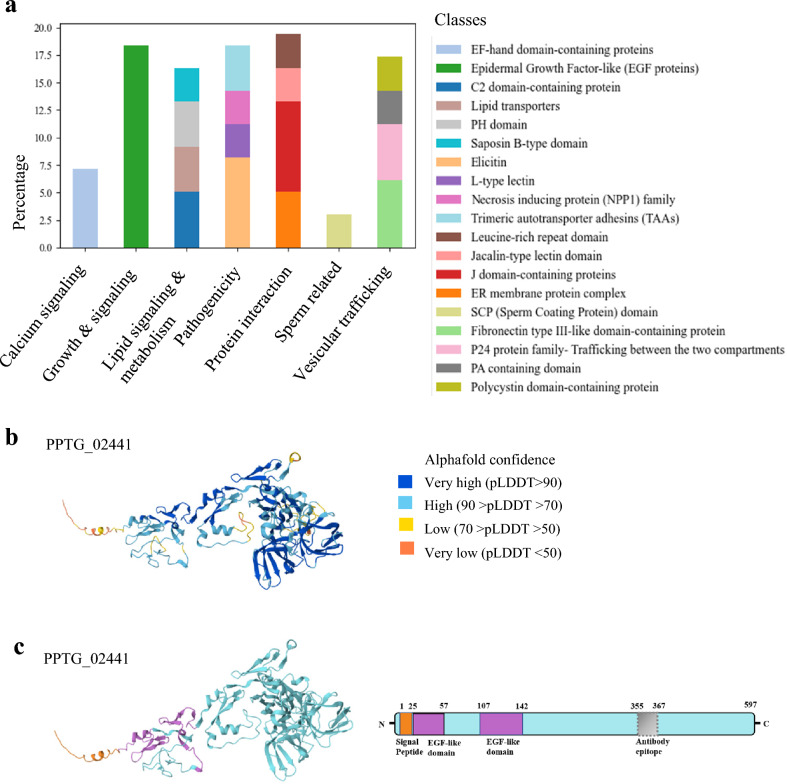
Annotation of other zoospore membrane proteins. (**a**) Distribution of other membrane-related proteins of zoospores presented as percentages. (**b**) AlphaFold model of PPTG_02441 with pLDDT score indicating accuracy of prediction for each residue. (**c**) Domain structure of the mastigoneme protein PPTG_02441, showing at least 2 EGFlike domains (in violet) and a signal peptide (orange).

Twelve percent of the proteins were categorized as other classified proteins. Within this category, notable groups include proteins associated with lipid-related functions such as sensing, transport, signalling, rafts, and metabolism (n = 30), *Phytophthora* pathogenicity (n = 26), growth and signalling processes (n = 19), protein-protein interactions (n = 23), and a smaller number related to sperm functionality (n = 3) (Fig. 6a).

The functional cluster of 30 proteins related to lipid sensing, transport, and signalling is consistent with the identification of proteins containing the SSD, mentioned above. These findings highlight that lipid uptake and regulation are crucial functions in *Phytophthora* zoospores. Among this group, proteins featured domains such as START (PPTG_06151, PPTG_16174) and PH (PPTG_16268, PPTG_14048, PPTG_12937, PPTG_12938), each known for distinct contributions to lipid-related activities in animals. The START domain specializes in the transport of lipids and sterol binding and is vital for the shuttling of these molecules across cellular compartments^73^. The PH domain acts in membrane-associated signalling pathways by binding to phosphoinositides, essential components in signal transduction mechanisms that involve lipids^74^.

The group of proteins implicated in oomycete pathogenicity (n=26) includes several putative elicitins and NPP1 proteins. Elicitins, conserved extracellular proteins in *Phytophthora* and *Pythium*, function as microbe-associated molecular patterns (MAMPs) to trigger defence responses in various plant species^75^. Interestingly, some elicitins are anchored to the oomycete PM rather than being fully secreted^75^. In particular, this may be the case for PPTG_17779, PPTG_07488, PPTG_13458, PPTG_08951 and PPTG_12308, which are annotated as elicitins and exhibit a GPI-anchor site. Elicitins are also known to bind sterols with varying affinities and can act as carriers, scavenging sterols from liposomes and plant membranes^76^. In *P. cinnamomi*, exogenous sterols downregulate the expression of two *β-cinn* elicitin genes as well as four *SCP* genes, supporting their role in sterol acquisition and virulence^72^. In *P. sojae*, sterol sensing is mediated by the membrane receptor SSRK1 in complex with elicitins^46^. Notably, we identified a putative SSRK1 homolog, PPTG_09207, predominantly detected in the *P. parasitica* cell body proteome. This raises the possibility that a similar elicitin–SSRK1-based sterol-sensing mechanism may operate in *P. parasitica*, potentially involving PPTG_09207 at the cell body level. On the other hand, analysis of the NPP1 proteins (here PPTG_15231, PPTG_08017 and PPTG_07664), which are implicated in causing necrosis in plant leaves and roots and are noted for being secreted, reveals that these proteins primarily feature a signal peptide, as previously documented^77^. The presence of these secreted proteins in membrane extracts may reflect their peripheral association with the plasma membrane via protein–protein interactions. Alternatively, their detection could result from co-purification artefacts, such as contamination of the membrane fraction with non-membrane proteins during sample preparation, particularly during membrane isolation steps.

Within the category of other classified proteins, another subgroup (n=18) comprises proteins involved in growth and signalling, mostly characterized by cysteine-rich EGF-like domains. This group included the three known mastigoneme proteins^9^ (PPTG_07238, PPTG_02441, PPTG_03648) that exhibit a signal peptide, three to four EGF-like domains, and no transmembrane domains. These proteins have been speculated to participate in mechanosensation and motion regulation in flagellated species within the Stramenopile taxon^9,78^. Figure 6b,c presents the 3D structure and the domain composition of PPTG_02441.

Among the 23 proteins involved in protein-protein interactions, we identified four putative leucine-rich repeat (LRR) domain-containing proteins (PPTG_07054, PPTG_07055, PPTG_00288, PPTG_12191). Although their specific functions are unclear, LRR domains are typically found in proteins involved in protein-protein interactions, including tyrosine kinase receptors and cell-adhesion molecules^47^.

Finally, three proteins (PPTG_08737, PPTG_00570, PPTG_08378) were annotated as harbouring a sperm coating domain (SCP, or CAP) and a signal peptide. CAP superfamily proteins are ubiquitous across all kingdoms of life, predominantly appearing in secreted forms, and play diverse roles in immune defence in plants and mammals, sperm maturation and fertilization, and fungal virulence^79^. Within the sperm context, certain CAP protein subfamilies modulate potassium channel activity, contributing to their functions in sperm maturation and motility^80,81^. The *P. parasitica* proteins may similarly influence flagellate zoospores, affecting ion transport mechanisms and ultimately motility.

### Flagella versus cell body variation

**Fig. 7:**
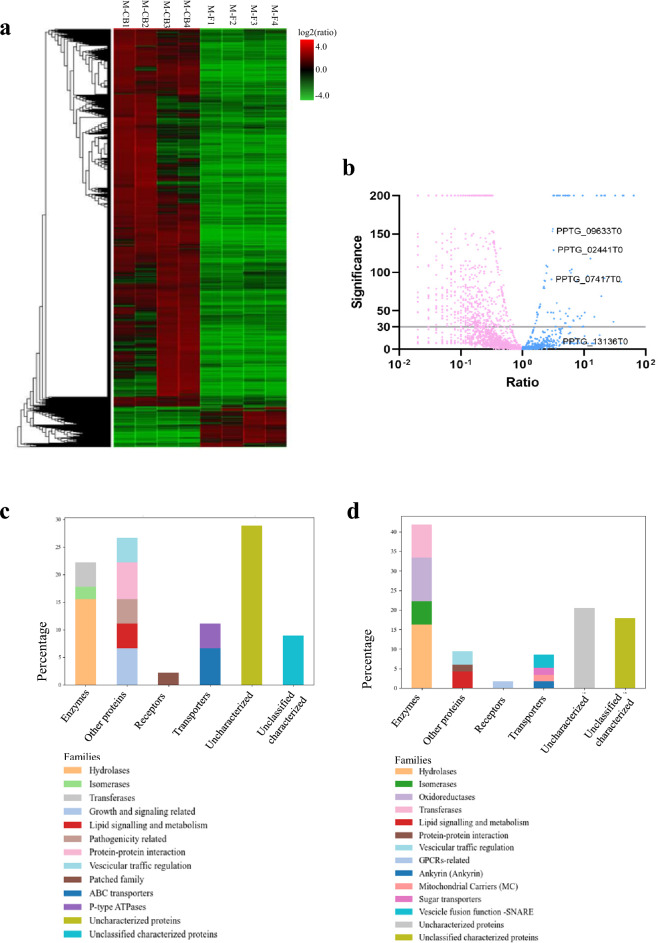
Distribution of proteins with significant differences in abundance between the four replicates of cell body and flagella samples. (**a**) Heatmap showing the proteins more abundant in the cell body (M-CB1.1 to M-CB4.1) or flagella replicates (M-F1.1 to M-F4.1). A clustering of M-CB and M-F samples is presented in Supplementary Fig. 5. (**b**) Volcano plot of the different quantified proteins highlighting the proteins of interest that are more abundant in the flagella replicates. PPTG_13136 was not differentially distributed according to the quantification method adopted. (**c**) Membrane protein families more abundant in the flagella fraction. (**d**) Membrane protein families more abundant in the cell body fraction.

A quantitative analysis was performed to compare M-CB and M-F fractions and showed that 785 proteins, out of the 6516 proteins initially identified by PEAKS, were differentially enriched between the cell body and flagella (Fig. 7a) (Supplementary Dataset File 2). Of these 785 proteins, 710 proteins were more abundant in cell body fractions (ratio < 0.5), while only 75 proteins were more abundant in the flagella (ratio > 2). Using the same filtering criteria as for the whole repertoire (GPI, signal peptide, and transmembrane predictions), we identified 137 membrane-associated proteins in the cell body fraction, and 45 membrane-associated proteins in the flagella fraction (which corresponds to more than half of the proteins more abundant in the flagella). Unsurprisingly, M-CB exhibited a broader diversity and higher number of membrane proteins compared to M-F, where the protein repertoire is naturally limited—especially for membrane proteins—as commonly observed in other flagellated eukaryotes^20,82^.

Compared to M-F (Fig. 7c), M-CB (Fig. 7d) was notably enriched in enzymes associated with trafficking and metabolic processes. Specifically, transferases constituted 7.3% of M-CB proteins versus 4.4% in M-F, and oxidoreductases were present exclusively in M-CB. Regarding transporters, M-F contained 3 ABC transporters (6.7%), while M-CB had only one. Likewise, M-F included 2 P-type ATPases (4.4%), whereas M-CB featured a single F-type ATPase. Despite this lower representation in common transporter classes, M-CB displayed a greater diversity of transporter families absent from M-F, such as ankyrin repeat-containing transporters, mitochondrial carriers, sugar transporters, and SNARE proteins. In contrast, M-F was characterised by a substantially higher proportion of uncharacterized proteins (28.8% vs. 17.5% in M-CB) and a larger fraction of other Classified proteins (26% vs. 8.7%). Within this category, M-F exhibited a wider array of proteins implicated in growth and signalling, also including the three mastigoneme proteins absent in M-CB—considered as quality control for quantitative analyses—as well as a pathogenicity-related protein uniquely found in M-F, the effector PPTG_07664^83^.

Interestingly, lipid sensing and motility regulation functions were prominently enriched in the M-F fraction. Among the proteins more abundant on the flagellar membrane were the sterol-sensing domain-containing protein PPTG_07417, with a 2.97-fold increase; the ABC transporter PPTG_08986, identified as an ABCA subfamily member involved in lipid trafficking^84,85^; and two putative lipases, PPTG_01586 and PPTG_11537. These observations suggest there may be a specific mechanism for sterol/lipid sensing and uptake, regulated at the flagellar level, that warrants further investigation. Finally, the most represented protein among the 1069 identified proteins, namely the Na^+^/K^+^-ATPase PPTG_09633, was also more abundant in the M-F fraction (Fig. 7b).

A subset (30 %) of the proteins enriched in the M-F fraction were not predicted to be membrane associated. Among these, we identified proteins with recognized roles in signalling chemotaxis in *Phytophthora*, including a G-protein α-subunit (PPTG_13453) and β-subunit (PPTG_18378)^16,69^. Interestingly, we also found the protein PPTG_11388, which harbours a VWA domain that mediates adhesion. This copine-like protein is very similar to CPNA in *Dictyostelium*, which is known to be involved in adhesion and chemotaxis^86^. Finally, we also found flagellar structural proteins such as α and β-tubulins, and centriole and basal body proteins^87^. This is consistent with the observation that pieces of axonemes and axonemal protein remain associated with the flagellar membrane after non-ionic detergent extraction, reflecting sites of tight association between the axoneme and the flagellar membrane^37^.

While the quantification results highlight some minor differences between M-CB and M-F, the apparent differences may have been reduced because: (i) the relative abundance of flagellar proteins may have been underestimated due to their synthesis in the cell body and transient accumulation at diffusion barriers before transport to the flagella (as seen with the mastigoneme protein PPTG_02441); and (ii) proteins missing a peptide area value in one replicate were excluded from differential quantification, and this included proteins such as PPTG_13136 and PPTG_13761﻿ that appeared more abundant in M-F and were confirmed by immunohistochemistry to be mostly localized on the flagella. Nevertheless, these results indicate a possible specialized role for the flagellar membrane in signal transduction and environmental response , emphasizing its functional adaptation beyond just motion. This aligns with findings in other flagellates like *Chlamydomonas*, where the flagellar membrane, though continuous with the cell’s PM, exhibits distinct protein variations linked to sensory functions^88^.

## Conclusions

The study posed three primary questions aimed at uncovering the protein repertoire in the PM used by zoospores to perceive and respond to external stimuli, and to drive their motion toward host plants:

I. What is the overall PM protein repertoire used by zoospores during signal perception and motion adaptation? Our results revealed new insights into how *Phytophthora* zoospores may use their PM protein repertoire for sensing and motility, particularly for sterol recruitment and ion flux variations.
II. Are there novel components within the repertoire that reveal new mechanisms of zoospore sensing? PPTG_13136 and PPTG_13761 emerged as potential members of a new ciliary protein family, conserved across 90 flagellated or ciliated organisms. These proteins may play a role in regulating flagellar motion, and the presence of a conserved voltage sensor domain suggests their potential involvement in ion-sensing processes. Although further investigation is needed to confirm their precise roles in zoospore sensing and motility, immunolocalization on *P. parasitica* zoospores revealed PPTG_13136 was predominantly localized on a single flagellum, strongly implying potential involvement in flagellar motility regulation.
III. To what extent are molecular sensors heterogeneously distributed in a zoospore? Our analysis identified an uneven distribution of molecular sensors, including the sterol-sensing protein PPTG_07417 and the S4 voltage domain-containing protein PPTG_13136. This suggests a possible polarization of sensory functions in zoospores that might use flagella both as sensory and motile organelles. This polarization likely plays a key role in regulating ion and lipid homeostasis, and sensing, transport, and signalling processes. These findings offer valuable insights into the role of flagella in zoospore motility and directional behaviour.

## Supplementary Information

Supplementary Dataset File 1.

Supplementary Dataset File 2.

Supplementary Data and Figures.

## Data Availability

The datasets generated and analysed during the current study are available in the PRIDE repository, https://www.ebi.ac.uk/pride/archive/projects/PXD059087.
