# Supplementary material for: Membrane proteome analysis identifies key components of sensing in Phytophthora parasitica zoospores
Source: Sci Rep. 2025 Jul 2;15:23500. doi: 10.1038/s41598-025-08701-z (PMC12223298; doi:10.1038/s41598-025-08701-z)
Supplement: Supplementary file 3 — Supplementary Data and Figures. [file 41598_2025_8701_MOESM3_ESM.pdf]

Liquid chromatography and mass spectrometry analysis

M-F and M-CB samples were fractionated by electrophoresis under reducing conditions into one-dimensional Sodium Dodecyl Sulfate-polyacrylamide (10%) gels. Four biological replicates of each fraction were loaded into a gel (5 µg/µL), subjected to electrophoresis (100V, 60 min) and later stained with Coomassie blue (Thermo Scientific PageBlue Protein Staining Solution). Gel tracks corresponding to biological replicates were sectioned into several bands. Each band was then cut into 1 mm^3 cubes and subjected to a washing process using water and acetonitrile (ACN). Subsequently, the gel pieces were reduced with 10 mM dithiothreitol in 50 mM ammonium bicarbonate for 30 min at 56°C. Following reduction, the samples were alkylated with 55 mM iodoacetamide in 50 mM NH_4HCO_3 for 20 min at room temperature in the dark.

The gel cubes were washed with 50 mM NH_4HCO_3 for 10 min, followed by a 15-min wash with acetonitrile (ACN), and then dried for 2 min in a SpeedVac (Savant, Apeldoorn, Netherlands). The cubes were subsequently incubated overnight at 37°C in a solution containing 25 mM NH_4HCO_3, 5 mM $CaCl_2$, and 12.5 ng/µL sequencing-grade modified trypsin (V5111; Promega, Madison, WI, USA)[18]. Enzymatic digestion was terminated by adding 5% formic acid (FA). The resulting peptides were extracted by washing the cubes twice with ACN. The samples were then dried for approximately 2 hours and desalted using OMIX C18 pipette tips (100 µL, A57003100) (Agilent, Santa Clara, CA, USA).

Samples were analyzed using a nanoUHPLC system (nanoElute) coupled to a TimsTOFpro mass spectrometer (Bruker Daltonics, Germany). Ten microliters of each sample were injected and separated on a reverse-phase C18 column with an integrated CaptiveSpray Emitter (75 µm ID x 250 mm, 1.6 µm, Aurora Series with CSI, ionOpticks, Australia) at a flow rate of 200 nL/min. The mobile phase consisted of 0.1% FA in water (Phase A) and 0.1% FA in ACN (Phase B). The liquid chromatography gradient started at 2% Phase B, ramping to 5% B in 1 min, then to 13% B in 18 min, followed by 22% B in 11 min, and finally to 95% B in 3 min, held for 7 min.

The TimsTOFpro mass spectrometer was operated with the CaptiveSpray nano-electrospray ion source. The source temperature was set to 180°C, with a spray voltage of 4500 V. Nebulizer gas (nitrogen) was supplied at 0.4 bar, and dry gas (nitrogen) was set at a flow rate of 3.0 L/min. MS and MS/MS data were acquired in positive polarity using Parallel Accumulation-Serial Fragmentation (PASEF)[19] Data Dependent Acquisition (DDA) mode, with 10 PASEF MS/MS

scans per cycle and a 100% duty cycle (Parallel Accumulation TIMS, EP3054473A1) (Parallel Accumulation TIMS, US9683964B2)[20]. Peptides were detected over a mass range of 100 to 1700 m/z, with a target intensity of 20,000 and an intensity threshold set at 2500. The collision energy was ramped linearly as a function of mobility, from 59 eV at 1/K0 = 1.3 Vs/cm² to 20 eV at 1/K0 = 0.7 Vs/cm² (TimsControl version 2.0.53.0).

The acquired DDA spectra were initially examined using Data Analysis software (version 5.3, Bruker Daltonics, Germany). Subsequently, the data were processed with PEAKS Studio (version Xpro, Bioinformatics Solutions) against the proteome predicted from the *P. parasitica* 310 genome[21]. The PEAKS identification search allowed for one missed cleavage, with carbamidomethylation set as a fixed modification and methionine oxidation as a variable modification. Contaminants were removed, with a parent mass error tolerance set to 20 ppm and a fragment mass error tolerance of 0.02 Da. Only proteins identified with an FDR of 1%, with at least one unique peptide, were selected.

Relative quantitative analysis of proteins was performed using the PEAKS Q label-free quantification method in PEAKS. This method, similar to the approach described by MaxQuant for high peptide identification rates and proteome-wide protein quantification[22], was applied to all four biological replicates for both groups M-F and M-CB. According to the method indications, for quantification results, only proteins with an FDR of less than 1% and a fold change greater than 2 were selected. Peptides considered for quantification had to be identified in both groups and detected in at least two samples per group, with quantification requiring a minimum of one peptide.

a

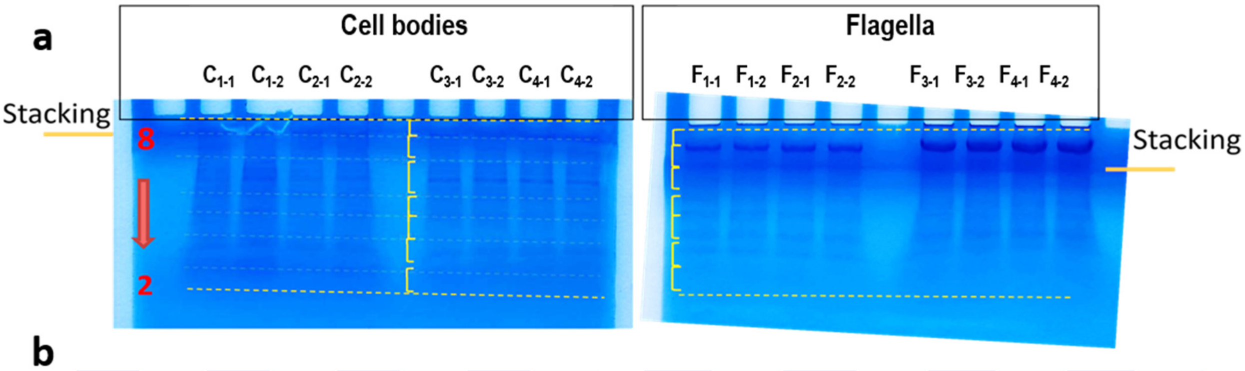

b

Lane C_{1-1}	Lane C_{1-2}	Lane C_{2-1}	Lane C_{2-2}	Lane C_{3-1}	Lane C_{3-2}	Lane C_{4-1}	Lane C_{4-2}
C1-1-8	C1-2-8	C2-1-8	C2-2-8	C3-1-8	C3-2-8	C4-1-8	C4-2-8
C1-1-7	C1-2-7	C2-1-7	C2-2-7	C3-1-7	C3-2-7	C4-1-7	C4-2-7
C1-1-6	C1-2-6	C2-1-6	C2-2-6	C3-1-6	C3-2-6	C4-1-6	C4-2-6
C1-1-5	C1-2-5	C2-1-5	C2-2-5	C3-1-5	C3-2-5	C4-1-5	C4-2-5
C1-1-4	C1-2-4	C2-1-4	C2-2-4	C3-1-4	C3-2-4	C4-1-4	C4-2-4
C1-1-3	C1-2-3	C2-1-3	C2-2-3	C3-1-3	C3-2-3	C4-1-3	C4-2-3
C1-1-2	C1-2-2	C2-1-2	C2-2-2	C3-1-2	C3-2-2	C4-1-2	C4-2-2

Lane F_{1-1}	Lane F_{1-2}	Lane F_{2-1}	Lane F_{2-2}	Lane F_{3-1}	Lane F_{3-2}	Lane F_{4-1}	Lane F_{4-2}
F1-1-8	F1-2-8	F2-1-8	F2-2-8	F3-1-8	F3-2-8	F4-1-8	F4-2-8
F1-1-7	F1-2-7	F2-1-7	F2-2-7	F3-1-7	F3-2-7	F4-1-7	F4-2-7
F1-1-6	F1-2-6	F2-1-6	F2-2-6	F3-1-6	F3-2-6	F4-1-6	F4-2-6
F1-1-5	F1-2-5	F2-1-5	F2-2-5	F3-1-5	F3-2-5	F4-1-5	F4-2-5
F1-1-4	F1-2-4	F2-1-4	F2-2-4	F3-1-4	F3-2-4	F4-1-4	F4-2-4
F1-1-3	F1-2-3	F2-1-3	F2-2-3	F3-1-3	F3-2-3	F4-1-3	F4-2-3
F1-1-2	F1-2-2	F2-1-2	F2-2-2	F3-1-2	F3-2-2	F4-1-2	F4-2-2

Figure of Supplementary Data 1. Coomassie-stained gels of proteins from M-F and M-CB samples.

(a) The four biological replicates of M-CB (C_1 to C_4; left gel) and M-F (F_1 to F_4, right gel) were analysed in two technical replicates (C_{1-1}, C_{1-2}, C_{2-1}, C_{2-2}, C_{3-1}, C_{3-2}, C_{4-1}, C_{4-2}; and F_{1-1}, F_{1-2}, F_{2-1}, F_{2-2}, F_{3-1}, F_{3-2}, F_{4-1}, F_{4-2}, respectively). Seven bands per lane (numbered from 2 to 8) were recovered (n=112). (b) Gel band preparation and LC-MS analysis were performed on one of the two technical replicates (blue cells, n = 56), with the other replicate retained for storage.

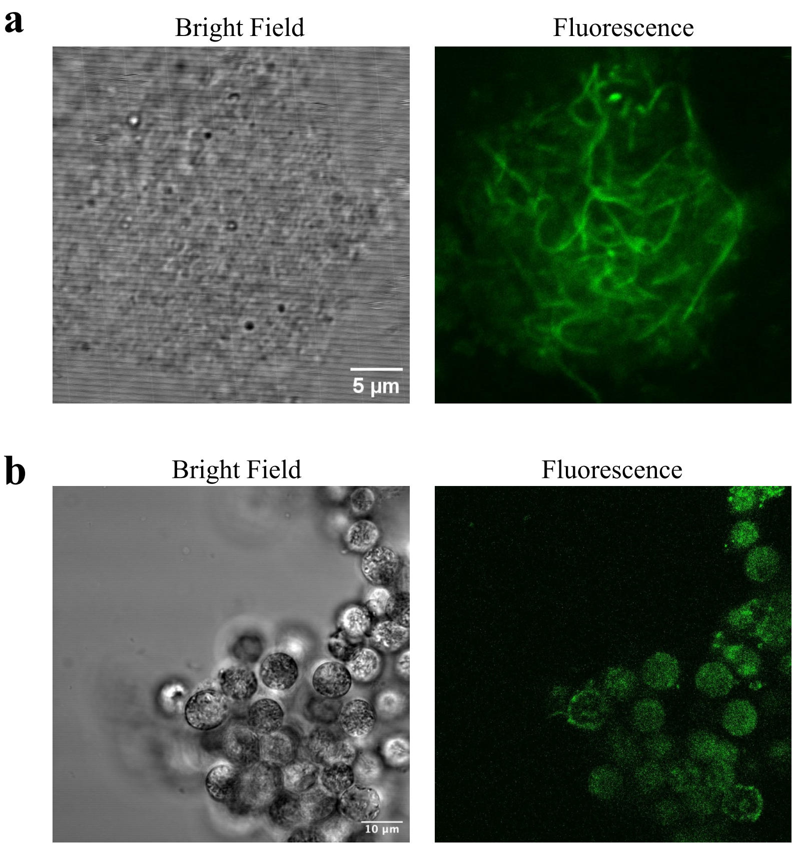

Supplementary Figure 1. Supplementary images of separated flagella and cell bodies.

(a,b) Bright field (left) and Fluorescence (right) views of Tubulin Tracker Green labelling of flagella and cell body fractions respectively. Images acquired with a Zeiss LSM 880 confocal microscope.

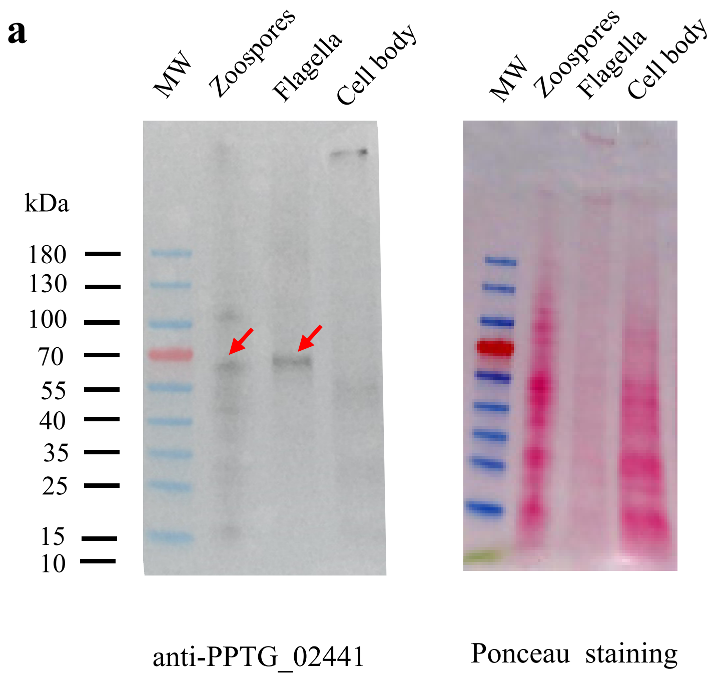

Supplementary Figure 2. Original immunoblot analysis using the anti-mastigoneme protein PTG_02441.

(a) The mastigoneme protein was detected at the expected calculated molecular weight (64kDa). Red arrows indicate the corresponding bands in zoospore and flagellum samples.

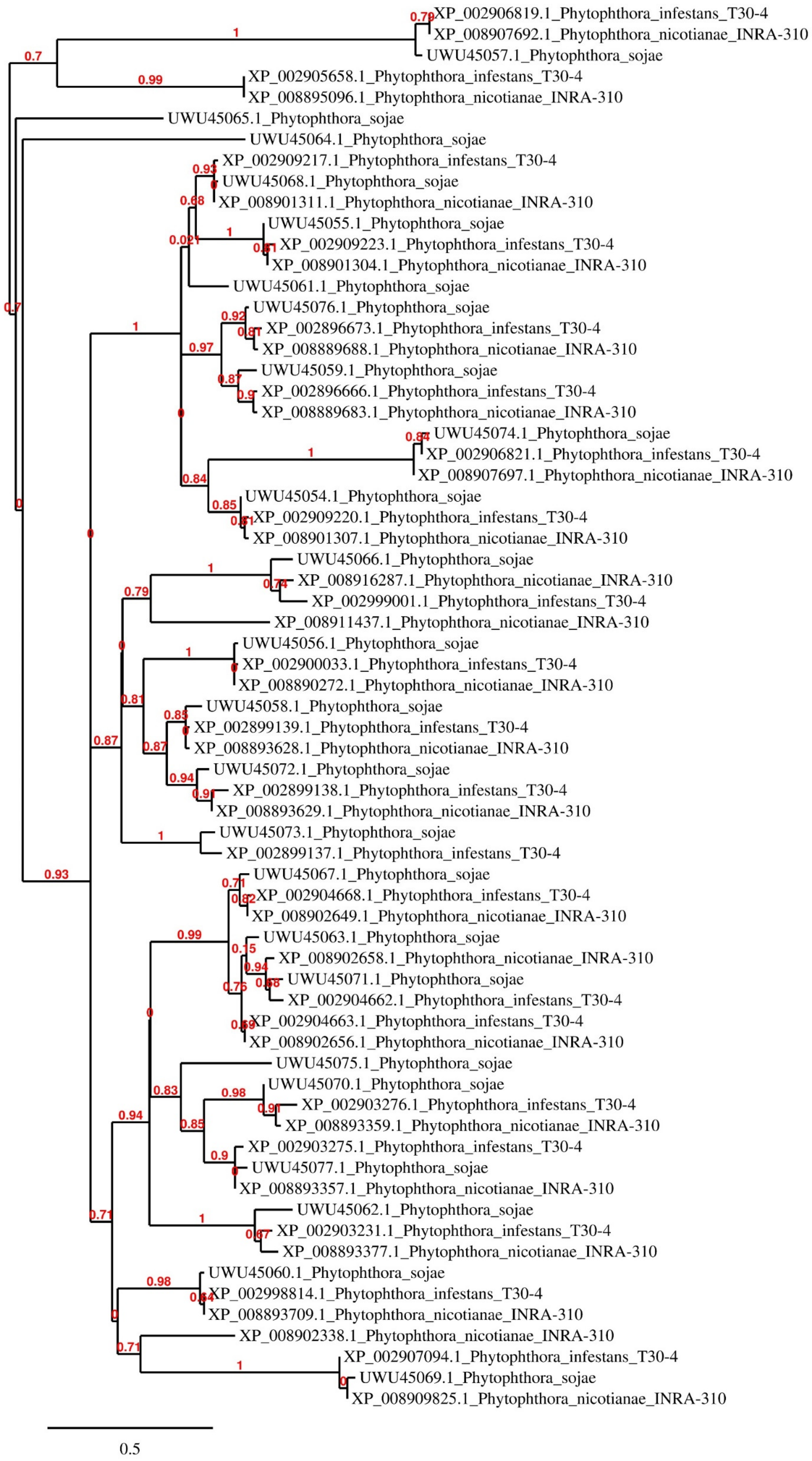

Supplementary Figure 3. Phylogenetic analysis of leucine-rich repeat receptor-like kinases.

Homologies to *P. sojae* proteins annotated as transmembrane leucine-rich repeat receptor-like kinases were search within *P. parasitica* and *P. infestans* proteomes performing a BLAST search. The *P. parasitica* proteins annotated as tyrosine kinase-like (PPTG_14643-XP_008909825.1, PPTG_16352-XP_008911437.1, PPTG_09207-XP_008902338.1, PPTG_01724-XP_008893359.1, PPTG_01987-XP_008893629.1, and PPTG_00076- XP_008889688.1), cluster closely with their *P. sojae* homologs.

a

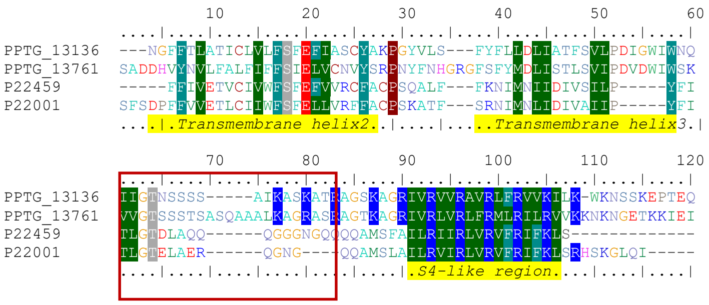

b

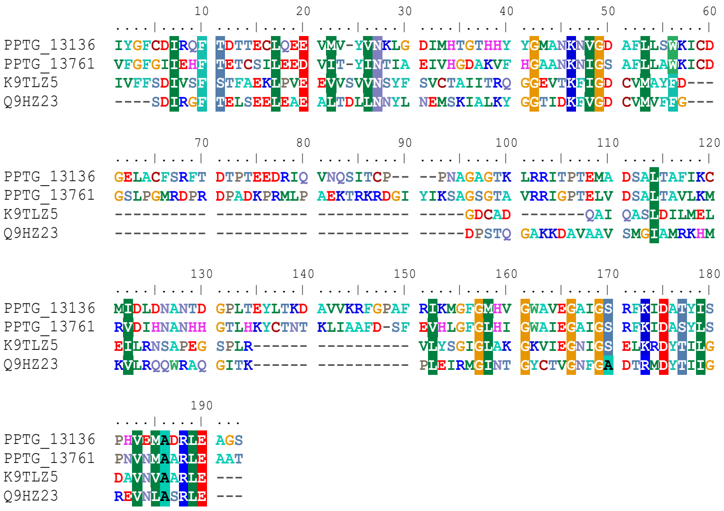

c

Phylum/Super group	Class/Clade	Protein number
Stramenopiles	Oomycetes	2 to 4
	Pelagophytes	2
	Diatoms	1
Alveolata	Apicomplexans	1 to 2
	Ciliates	6 to 16
	Dinoflagellates	3
Chlorophyta	Green algae	1
Haptista	Haptophytes	1

d

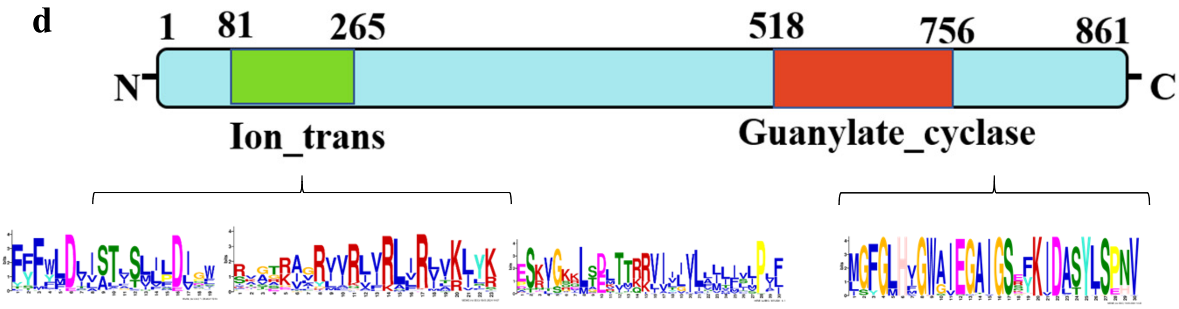

Supplementary Figure 4. Sequence properties of PPTG_13136 and PPTG_13761.

(a) Alignment using ClustalW multiple alignment in BioEdit, of a part of the N-terminal Ion_trans PFAM domain of PPTG_13136 and PPTG_13761, compared with two human potassium voltage-gated channels, subfamily A member 4 (P22459) and member 3 (P22001). Shaded amino acids indicate AA homology; yellow boxes below sequences indicate the position of the TMD domains and of the matching with the helical segment S4, the voltage sensor of the voltage-dependent gated channel. (b) Alignment of the nucleotide cyclase domain of PPTG_13136 and PPTG_13761 with two bacterial photoactivated adenylyl cyclases (K9TLZ5 and Q9HZ23). (c) Table showing the number of proteins with domain organizations similar to PPTG_13136 and PPTG_13761 across different phyla. The highest BLAST scores are predominantly found in flagellate organisms, suggesting a role for these proteins in motility. The variability in protein numbers likely reflects the functional and structural complexity of each group, with ciliates exhibiting higher numbers (6-16), possibly due to the greater demands of their multiple ciliary structures. (d) Analysis of conserved domains in PPTG_13136. The proteins share with other similar bimodal proteins different conserved domains, corresponding here (from left to right) to the third transmembrane helix (4.8e-773 e-value), the S4 voltage sensor (4.3e-874 e-value), the fourth transmembrane helix (1.1e-1099 e-value) and part of the guanylate cyclase domain (4.5e-2375 e-value). Analysis was conducted using the MEME Suite (MEME Suite, Bailey et al., 2015).

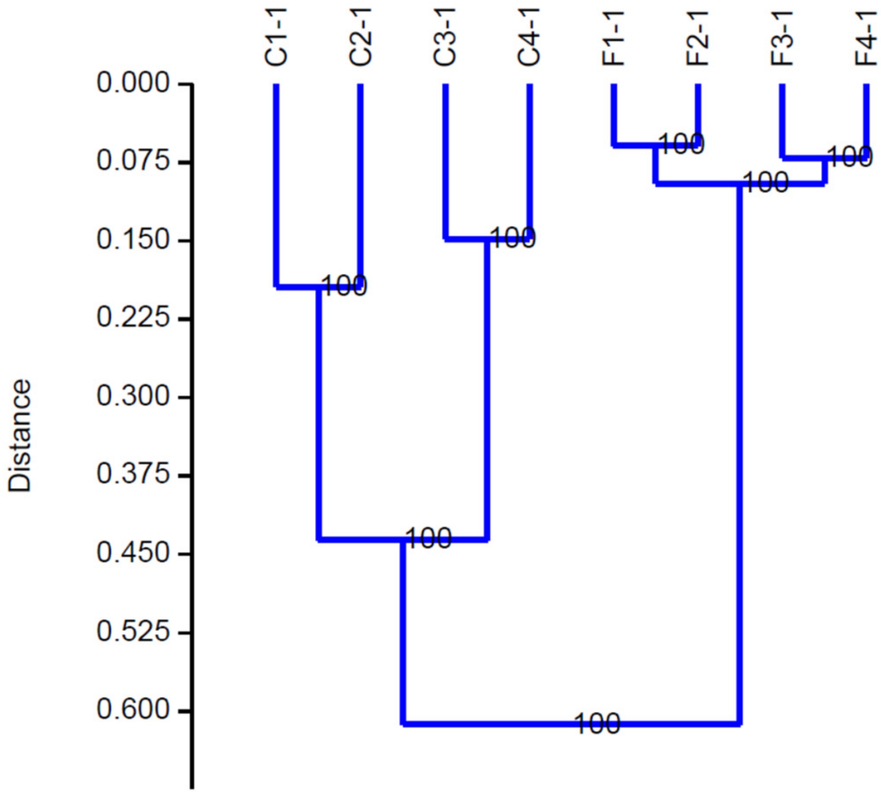

Supplementary Figure 5. Hierarchical clustering of cell body and flagellum replicates from the proteomic analysis.

Hierarchical clustering of protein abundance profiles in cell body (M-CB) and flagellum (M-F) replicates. Clustering was performed on the sum of the areas of the three most abundant peptides for each of the 785 proteins identified as differentially abundant between compartments. The resulting dendrogram reveals two distinct clusters: one grouping the four cell body replicates and the other grouping the four flagellum replicates. Distance values represent consensus clustering from 1,000 iterations using the PAST software.
